# Differences in Anatomy and Kinematics in Asian and Caucasian TKA Patients: Influence on Implant Positioning and Subsequent Loading Conditions in Mobile Bearing Knees

**DOI:** 10.1155/2014/612838

**Published:** 2014-11-20

**Authors:** Allan Maas, Tae Kyun Kim, Rolf K. Miehlke, Thomas Hagen, Thomas M. Grupp

**Affiliations:** ^1^Aesculap AG Research & Development, Am Aesculap-Platz, 78532 Tuttlingen, Germany; ^2^Joint Reconstruction Center, Seoul National University Bundang Hospital, Seongnam 463-707, Republic of Korea; ^3^Knee Division, The Rhine-Main Centre for Joint Diseases, Wilhelmstraße 30, 65183 Wiesbaden, Germany; ^4^Knee Division, The Muensterland Centre for Joint Diseases, Buelt 13, 48143 Muenster, Germany; ^5^Ludwig Maximilians University Clinic for Orthopaedic Surgery, Campus Grosshadern, Marchioninistraße 15, 81377 Munich, Germany

## Abstract

The objective of our study was to determine the mechanical stress conditions under tibiofemoral loading with an overlay of knee kinematics in deep flexion on two different mobile bearing designs in comparison to in vivo failure modes. This study investigates the seldom but severe complication of fatigue failure of polyethylene components at mobile bearing total knee arthroplasty designs. Assuming a combination of a floor-based lifestyle and tibial malrotation as a possible reason for a higher failure rate in Asian countries we developed a simplified finite element model considering a tibiofemoral roll-back angle of 22° and the range of rotational motion of a clinically established floating platform design (e.motion FP) at a knee flexion angle of 120° in order to compare our results to failure modes found in retrieved implants. Compared to the failure mode observed in the clinical retrievals the locations of the occurring stress maxima as well as the tensile stress distribution show analogies. From our observations, we conclude that the newly introduced finite element model with an overlay of deep knee flexion (lateral roll-back) and considerable internally rotated tibia implant positioning is an appropriate analysis for knee design optimizations and a suitable method to predict clinical failure modes.

## 1. Introduction

Evaluating the success of total knee arthroplasty (TKA), the incidence of failure due to implant fracture is a relatively rare condition [1–3]. Sadoghi et al. [3] reported the common causes for revision surgery in TKA to be aseptic loosening (29.8%), septic loosening (14.8%), pain without any other reason (9.5%), wear (8.3%), instability (6.2%), implant breakage (4.7%), and periprosthetic fracture (3.0%). In this complication-based analysis out of 391,913 primary TKAs entered in the arthroplasty registers in Norway, Sweden, Denmark, Finland, Australia, and New Zealand from 1979 to 2009, 36,307 revisions were reported, a rate of 9.3% leading to a relative value of 0.44% revisions due to implant fracture. In an analysis of 3198 revisions out of 41,223 primary knee arthroplasties with a 10-year follow-up captured in the Swedish Knee Arthroplasty Register, Robertsson et al. [1] identified implant fracture as a minor reason for revision in 0.94% of the cases. Based on the large cohort of primary knee arthroplasties a relatively rare incidence of 0.073%. Gøthesen et al. [2] extracted the causes of revision for 17,772 primary TKAs based on the Norwegian Arthroplasty Register between 1994 and 2009 and found an incidence for seven different implant designs an incidence for implant fracture from 0.0 to 0.3%.

It is the intention of mobile bearing knee designs to reduce the risk of structural material damage and fracture of the polyethylene gliding surfaces, offering the advantage of high mobility in combination with little constraint and low contact and subsurface stresses [4–7]. Analysing the contact stress situation in the tibiofemoral articulation, the majority of studies concentrate on level walking and stair climbing activities with related flexion angles between 0° and 90° [8, 9]. Only some finite-element-analysis (FEA) studies have focused on the tibiofemoral loading situation in deep flexion activities [4, 5] showing comparably high surface contact stresses due to design related lower conformity for more than 90° flexion [10]. Some recently published finite element studies analysed the effect of tibial malrotation on the polyethylene stress distribution and magnitude of fixed and mobile designs during high flexion in a simplified dynamic analysis setup [11, 12]. A limitation of these studies is the orientation of the femoral component in a neutral or constant position relative to the tibia throughout the complete flexion range from 0° to 135°. However, in normal knee kinematics, a medial pivot (MP) pattern combined with a lateral roll-back (RB) has been described [13–16], resulting in a substantial external rotation of the femur relatively to the tibia and a pronounced dorsal loading of the lateral aspect of the gliding surface.

Asian patients differ from Caucasian patients in anatomy [17–20], degree and patterns of deformities [21–24], and cultural aspects [25–27]. Because of cultural characteristics in Asia, such as a traditional floor-based lifestyle call for frequent high-flexion activities in daily living, Asian patients after TKA tend to have a substantially higher range of motion (ROM) compared to Caucasian patients [28–32]. Anatomy driven internally rotated positioning of the tibial tray in combination with a lateral roll-back of the femoral condyle in deep flexion [14, 16, 33] may lead to demanding loading conditions on the gliding surfaces of mobile bearing knee implants and subsequent fracture of the dorsolateral portion of the gliding surface.

## 2. Objectives

The objective of our study was to determine the mechanical stress conditions under tibiofemoral loading with an overlay of knee kinematics in deep flexion on two different mobile bearing designs, under clinically relevant implant positioning in comparison to in vivo failure modes found in retrieved implants.

**Figure 1:**
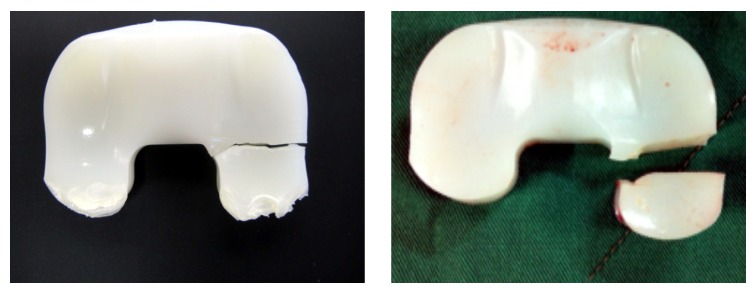
Clinical retrievals of the investigated FP system with polyethylene fracture of the lateral condyle.

As described above, failure of polyethylene inserts in mobile bearing total knee arthroplasty due to fatigue fracture as shown in Figure 1 is uncommon and reported rarely [34]. Although the failure rate of the herein investigated multidirectional floating (FP) system is comparatively low (0.02%, 54.422 implantations) in European countries, a two times higher failure rate in Asian countries was observed (0.04%, 20.577 implantations). Due to the fact that Asian countries have visibly lower overweight and obesity rates than European countries [35, 36] a higher failure rate caused by mechanical overloading of the components seems implausible.

Our hypothesis was that a combination of a higher range of active knee flexion and initially internally rotated tibia components may produce a loading scenario under which a polyethylene insert is exposed to tensile stress levels above the yield strength of the ultrahigh-molecular-weight polyethylene (UHMWPE) material as a result of bending the meniscal component over the posterior end of the tibia component. This study was performed to test the hypothesis, in other words, to determine whether the polyethylene insert is exposed to excessive overloading at a high-flexion angle in combination with a malrotated tibia plateau. In addition, an improved posterior stabilized rotating platform design (RPS) was also included in the present investigation to evaluate the effect of the modifications in a direct comparison.

## 3. Materials and Methods

### 3.1. Knee Systems

**Figure 2:**
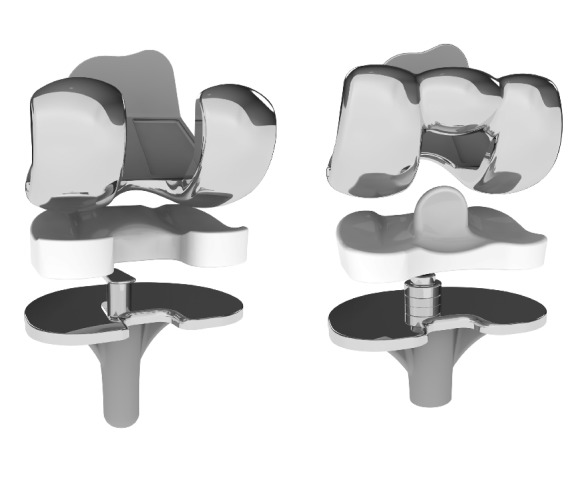
Analyzed systems, left image shows the FP TKR and right image shows the improved RPS system.

Two mobile bearing systems of the common size combination F4/T3 with an UHMWPE inlay of the lowest available height (10 mm) were compared in this study. The e.motion FP multidirectional floating TKA system (Aesculap, Tuttlingen, Germany), where posterolateral failure of the meniscal component was observed in 22 cases (reported failure cases until 11/2013) and the e.motion Pro posterior stabilized rotating platform (Aesculap, Tuttlingen, Germany) system (Figure 2) without any reported failures.

**Figure 3:**
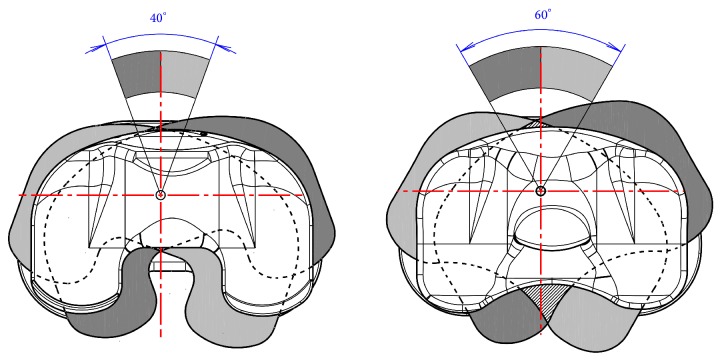
Range of rotational motion provided by the system (left: FP, right: RPS design).

Both systems have an integrated mechanical stop to avoid a dislocation (spin-out) of the polyethylene insert. The allowed range of rotational motion at the FP system measures ±20° whereas the RPS system allows a rotation of ±30° until the movement is restrained (Figure 3). For the geometrical models original manufacturing CAD data were used. For both of the analysed systems four CAD models were built starting from a neutral position of the tibia component from 0° to a 5°, 10°, and 15° internally malrotated plateau (Figure 6).

### 3.2. Alignment

**Figure 4:**
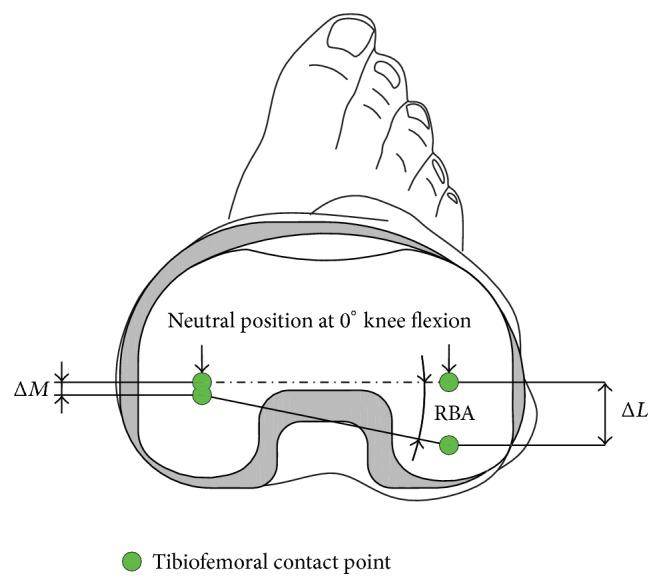
Schematic description of the used roll-back angle definition in relation to the gliding surface.

The roll-back angle (RBA; Figure 4) definition herein used is described as the relative posterior motion of idealized tibiofemoral contact points with flexion referred to their location at a neutral knee position at 0° knee flexion angle (KFA). The relative tibiofemoral rotation of the normal knee was investigated extensively by many researchers who also described a medial pivot behavior of tibiofemoral contact areas in higher knee flexion [37–40]. The results determined by Iwaki et al. [13] in a cadaveric study on 6 knees show a relative rotation of tibiofemoral contact points around a medial pivot (mean MP ± 1.5 mm) of 22.4° at 120° KFA [13]. Comparable results were found by Dennis et al. [41] (mean MP ± 1.94 mm) with an average relative RBA of 23.7° and by Asano et al. [42] (mean MP ± 6.9 mm) with a mean relative RBA of 23.8°. Leszko et al. [43] investigated Japanese female and male normal kinematics at even higher flexion angles and determined average maximum values for Japanese females (RBA 29.8° at 153° KFA). Japanese males reached slightly less maximum knee flexion (RBA 27.9° at 151° KFA).

Although only for a KFA of 110° Suggs [44] and Suggs et al. [16] found comparable contact kinematics of the normal knee and total knee arthroplasty with cruciate ligament retaining implant systems. The recently published study of Meccia et al. [45] included a group of 58 TKA patients, forty with fixed (34PS, 5PCR, and 1ACL-R) and 18 with mobile (5PS, 5PCR, and 8PCS) bearing designs. This cohort showed an average relative tibial rotation of 18.1° (min. 15°, max. 26.9°) at an average KFA of 114.8° (min. 90°, max. 147°).

Regarding cruciate substituting PS designs the exact tibiofemoral contact position at 120° KFA is hard to predict since it depends on the design which influences the motion after cam-post contact. There are only a few publications which are expedient for the current study. Victor et al. [46] performed a study on fifteen patients (eight with fixed cruciate retaining and seven with fixed cruciate substituting TKA) randomly chosen from a well selected group of 44 patients. Although they further described the relative position of the femur relative to the tibia only between 0° and 80° KFA, they published data which suggest roll-back behaviour between 120° and 140° for the cruciate retaining system as described before. The cruciate substituting knee group showed a smaller RBA and a different pivot point. At the end of the regarded flexion cycle at 80° KFA an anterior movement of the medial and a posterior movement of the lateral contact region were observed. This behaviour suggests a rotation around the postmechanism. A similar behaviour was also reported by Suggs [44]. They compared a group of US patients (8 females and 12 males) to a group of south Korean (SK) patients (24 females) with fixed PS TKA and determined the relative movement of medial (M) and lateral (L) tibiofemoral contact points. Although the mean maximum range of active knee flexion was comparable (US: 113.3 ± 19.4°, SK: 112.5 ± 13.1°), the SK group showed a larger RBA at maximum KFA (mean ΔML = 14 mm) compared to the US patients (mean ΔML = 3.1 mm). Assuming a medium condylar distance of 42 mm the SK group showed an average relative tibiofemoral rotation of 18.4° with a fixed PS knee implant at mean 112.5° KFA.

Regarding the comparable values determined by different researchers, a simplified mechanical relationship between KFA and RBA may be used as a rule of thumb to roughly estimate the relative tibiofemoral rotation for different types of well implanted TKR designs which allow a rotational motion and the normal knee between 90° and 150° knee flexion of RBA ≈ KFA/5.5. In the present study this will result in a relative rotation of tibiofemoral contact points at 120° KFA of 21.8° or 22° as a rounded value.

**Figure 5:**
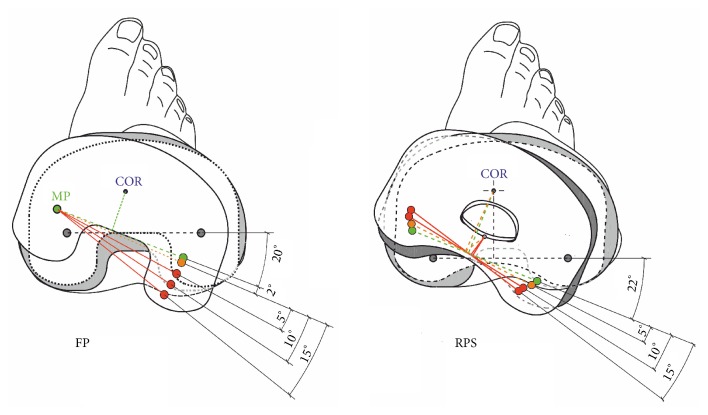
Comparison of the tibiofemoral contact points assumed for the analyzed scenarios at the FP (left) and RPS (right) TKA design at 120° knee flexion.

**Figure 6:**
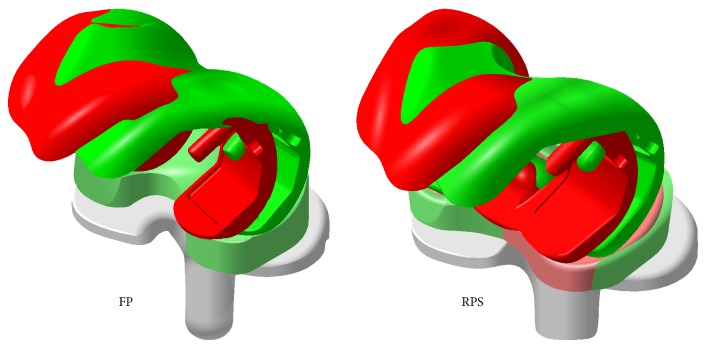
CAD geometry of neutral positioned (0°, green) and maximum malrotated (15°, red) situation, insert at the FP (left) TKA design is already full rotated at 0°.

To compare the two different systems at 120° KFA, 22° RBA was defined as a normal condition. With both of the systems relative displacement between meniscal and femoral component starts after hitting the mechanical stop. At a neutral oriented tibia component (0° internal malrotation) of the FP system the mechanism which allows a range of rotational motion of ±20° is already hit at 120° KFA resulting in a relative tibiofemoral rotation around a medial pivot of 2°. Each degree of internal tibia malrotation can be added directly on the relative rotation between femoral component and gliding surface. At the RPS system the mechanism which allows ±30° is not active with a neutral implanted tibia component. Relative displacement between meniscal and femoral component first occurs at a malrotation of the tibia component of 8° where the centre of rotation changes from the mechanical rotation axis of the RPS system to the cam-post contact point (Figure 5).

### 3.3. FE Model

**Figure 7:**
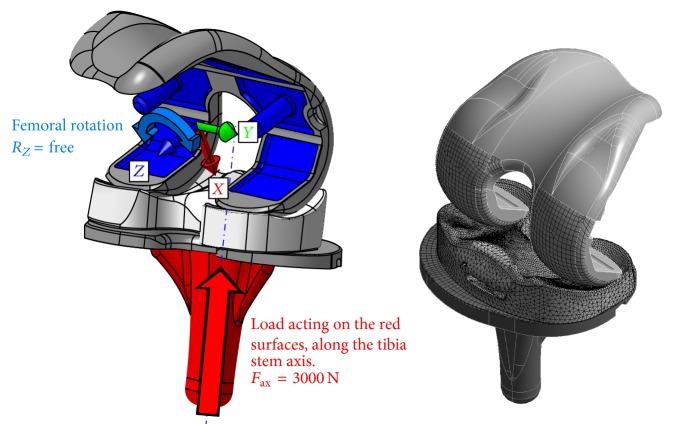
Description of the boundary conditions (left) and initial mesh of the FE model (right).

Eight CAD assemblies were transferred to the FE-software package (Ansys R15) to perform nonlinear static analyses of a single loading scenario. Comparable to the method used by Godest et al. [47], the femoral and tibial components as well as the rotation stop mechanism were modelled as rigid bodies. The meniscal component of each assembly was modelled as flexible body and was meshed using 10-noded tetrahedral structural solid elements (Ansys SOLID187) with a global element size of 1.5 mm. The proximal and distal contact areas including the surfaces in contact with the stop mechanism were resized to an initial element edge length of 0.75 mm and received an additional layer of contact shell elements of the same size and mechanical characteristics to represent contact and sliding between the parts (Ansys CONTA174). The rigid surfaces in contact were coated with the corresponding counterpart elements (TARGE170) using an element size of 1.5 mm. For all contact regions, a frictional behaviour with a friction coefficient of *μ* = 0.025 [48] was defined by using an augmented-lagrangian contact algorithm and a contact stiffness parameter FKN = 1. Degrees of freedom of the rigid bodies were restricted by nodal multipoint constraints (ANSYS MPC184). The tibia component was only free to move along its vertical stem axis while the femoral component was locked in all degrees of freedom except of the rotation around the vertical axis of the femoral component (Figure 7). This allows an optimized alignment of the components under load without influencing the relative position of the components regarding knee flexion angle or internal tibia rotation. The meniscal component had no additional restrictions. To improve the results, an adaptive meshing convergence criterion of 1% was added to the 1st principal stress maximum value (tensile stress) determined at the meniscal component.

### 3.4. Loads

Regarding the failure mode shown in Figure 1, overloading of the polyethylene component in an unfavourable position is the most likely reason and therefore high loads are expected at the meniscal component at a knee flexion angle of 120°. The recently published load data of Bergmann et al. [49] averaged to 75 kg patient weight (AVER75) and also for a patient weight of 100 kg (High100) deliver an axial load magnitude during a knee bend exercise between 1942 N (AVER75) and 3407 N (HIGH100) at a maximum knee flexion angle of 98°. For the present analysis series, a constant load of 3000 N was applied to the tibia component acting along the stems axis as shown in Figure 7.

### 3.5. UHMWPE Material Model

**Figure 8:**
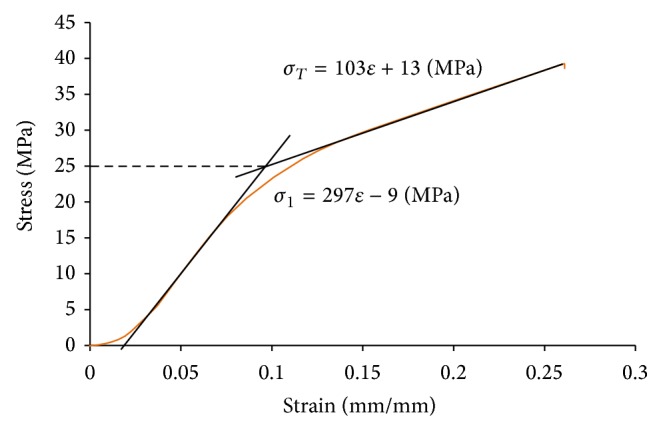
Example test data (orange curve) with bilinear material curve fit and determined material parameters.

The UHMWPE material model was generated using test data of an uniaxial test series on beta irradiated GUR1020 polyethylene samples as used for the gliding surfaces. An example curve of this series is shown in Figure 8. After the test series, the used setup was rebuilt inside the FE System and the determined parameters were verified by an analysis using equal conditions (element types, sizes, etc.) as in the present analysis series. The material model uses a bilinear formulation which accounts for plastic deformation behavior after exceeding a yield point. The rounded average value determined for the first Young's modulus was *E* = 297 MPa, followed by a tangent modulus of *E*_*T*_ = 103 MPa after reaching the *σ*_Yield_ = 25 MPa point. The materials Poisson's ratio of *μ* = 0.35 was chosen after performing a parametric FE-study on the above described FE model [50].

## 4. Results

**Figure 9:**
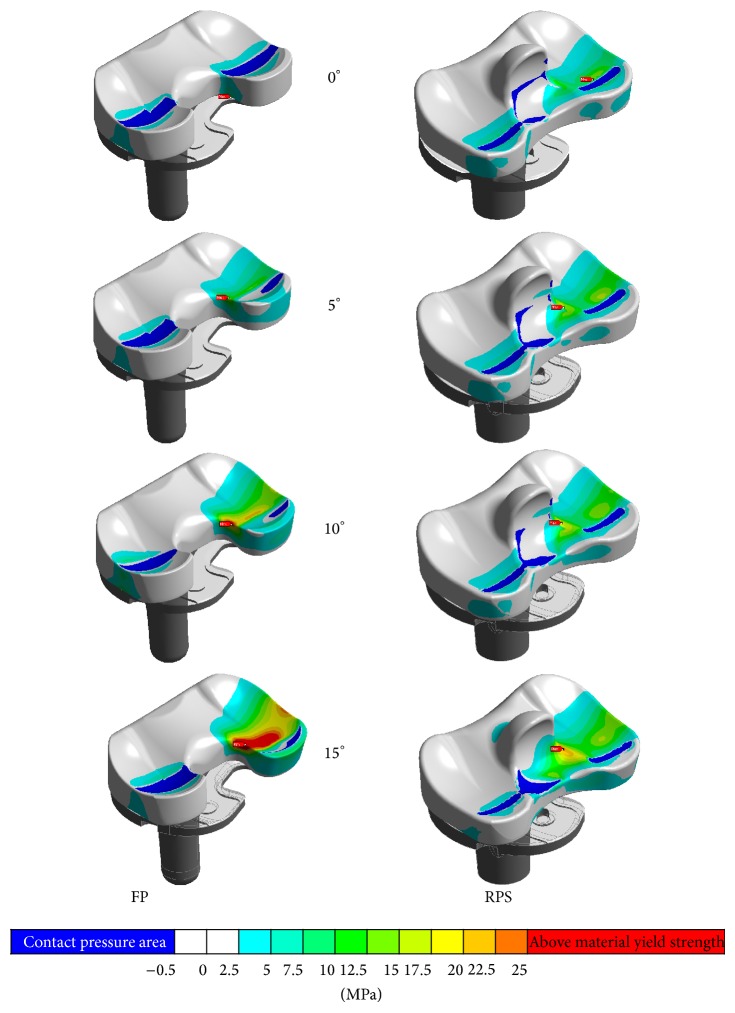
Tensile stress contour plots with marked locations of stress maxima at the FP (left column) and the RPS (right column) design at all analysed tibia rotations.

**Figure 10:**
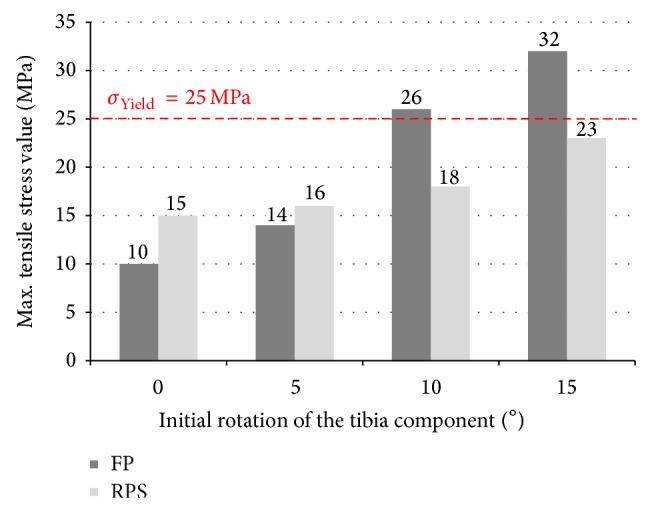
Summary of tensile stress maxima determined for both of the analysed systems.

All analysed FE models fulfilled the claimed 1% convergence criterion on tensile stress maxima applied to the meniscal component after a single mesh refinement step. At neutral tibia position the occurring maximum stress value at the FP system is 36% lower than the value determined for the RPS design (FP = 9.6 MPa versus RPS = 15 MPa); at a tibia malrotated position of 5° both systems are in the same range regarding the maximum tensile stress value (FP = 14 MPa versus RPS = 16 MPa). At a tibia rotation 10° off the neutral position the maximum stress value determined at the FP system is 44% higher than the value computed for the RPS system (FP = 26 MPa versus RPS = 18 MPa), first exceeds the material yield strength of 25 MPa and also a deformation of the lateral compartment is visible. At a malrotation angle of 15°, the FP geometry shows large deformations in the posterolateral region and a 40% higher maximum stress value compared to the RPS system which is still slightly below the materials yield strength (FP = 32 MPa versus RPS = 23 MPa). The tensile stress contour plots with marked location of the occurring stress maximum value are shown in Figure 9. The results are summarized in Figure 10.

## 5. Discussion

The objective of our study was to determine the mechanical stress conditions under tibiofemoral loading with an overlay of knee kinematics in deep flexion on two different mobile bearing designs, under clinically relevant implant positioning in comparison to in vivo failure modes on retrieved devices.

Based on the observation of a two times higher failure rate for fracture of the gliding surfaces of a multidirectional floating platform knee design in Asian countries compared to European countries, we formulated the hypothesis that a combination of anatomy driven internally rotated tibia components and a higher range of active knee flexion may produce a loading scenario which is able to generate tensile stress levels above the yield strength of the UHMWPE material.

Another possible reason for fracture of TKA gliding surfaces is implant overloading by overweight or obesity of the patients, but this factor was withdrawn because in Asian countries the rate of obesity is substantially lower than in European countries. The OECD Factbook [35] reports a percentage of obese population of 4.7% for South Korea, 3.2% for Japan, 3.4% for China, 2.8% for India, and 3.6% for Indonesia compared to 13.8% for Germany, 13.4% for France, 9.6% for Italy, 26.1% for the United Kingdom, 12.6% for the Netherlands, 14.4% for Belgium, 13.1% for Sweden, 8.0% for Norway, 12.7% for Austria, and 7.7% for Switzerland.

Also the body mass index (BMI) for TKA patients is in Asia lower than in Western countries.

Gandhi et al. [51] found in a cohort of 1876 White patients a mean BMI of 30.1 kg/m^2^, compared to 28.7 kg/m^2^ for Indian patients (*n* = 107). Allen et al. [52] reported a mean BMI of 32.1 kg/m^2^ for 324 White patients with hip or knee osteoarthritis and of 35.6 for 216 African American, whereas Kim et al. [53] described a mean BMI of 26.7 kg/m^2^ based on the epidemiology of 47,961 TKA patients given in the South Korean national registry. Siow et al. [36] found a mean BMI of 27.5 kg/m^2^ for 4713 Chinese patients, of 31.5 kg/m^2^ for 304 Malay patients, and of 30.2 kg/m^2^ for 315 Indian patients undergoing TKA in Singapore.

From our point of view the main influencing factor for insert failure is the significantly higher degree of active knee flexion in Asian patients in comparison to European patients. Kim et al. [30] reported for 66 South Korean TKA patients implanted with the e.motion FP design with a follow-up of 24 months a range of knee flexion of 140.1° ± 13° preoperatively (pre-op) and of 130.7° ± 9.0° postoperatively (post-op). For the same FP design implanted in Germany Geiger et al. [31] found in 60 patients with a follow-up of 24 months a knee flexion of 104.7° ± 18.9° pre-op compared to 119.3° ± 14.2° post-op and also Miehlke and Thiel [54] described for 125 TKA patients with 36 months follow-up an improvement in flexion ability from 108° pre-op to 122° post-op. Saragaglia et al. [55] reported for a cohort of 31 TKA patients with severe genu varum deformities in France treated with the FP design with a follow-up of 31 months a mean range of flexion of 116.9° ± 12.5° pre-op and of 114.1° ± 10.6° post-op. Seon et al. [56] found for the FP design an increase in active flexion from 115.8° pre-op to 127.1° post-op in a cohort of 100 South Korean TKA patients with a follow-up of 24 months and in a second independent series of 42 patients with 12 months follow-up 116.9° pre-op compared to 128.1° post-op [28].

It is well known that mobile bearing TKA designs have some advantages regarding wear, range of motion and they also excuse little deviations in implant positioning because they align automatically when compared to fixed-bearing designs [12, 57–62]. But even with the use of computer assisted navigation the tibial rotational alignment is challenging and highly variable [63]. As described the positioning tolerance of the implant system may be limited as shown in the present study and an initially internally rotated tibial plateau may cause high stresses at the meniscal component, when it comes to high knee flexion angles because of the integrated stop mechanism necessary to avoid dislocation of the insert.

**Figure 11:**
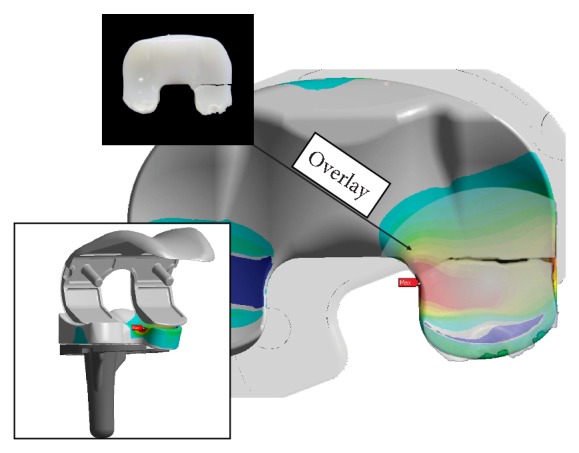
Semitransparent overlay of a retrieval image onto the FE-result of 15° malrotation at the FP system (patient data: Female/69, after 7.5 years).

The hypothesis that the assumed scenario might lead to polyethylene fracture seems to be plausible because the region of stress values exceeding the material yield strength and the region of failure observed at clinical retrievals [34] match quite well (see Figure 11). In this fictive loading scenario the stress level at the FP system becomes critical at a malrotation of 10° where the determined maximum tensile stress value first exceeds the material yield strength.

Compared to the analysed multidirectional floating platform TKA system the posterior stabilized rotating platform design is more tolerable regarding tibial malpositioning and the resultant maximum stress values did not exceed the yield strength of the UHMWPE material even with a malrotation of 15°.

## 6. Conclusion

From our observations, we conclude that the newly introduced finite element model with an overlay of deep knee flexion (lateral roll-back) and considerable internally rotated tibia implant positioning is an appropriate analysis for knee design optimizations and a suitable method to predict clinical failure modes.

In performing knee arthroplasty, the surgeon should be aware that an anatomy driven internal rotation of more than 10° may cause the risk of fracture of the dorsolateral portion of mobile bearing gliding surfaces due to loading conditions exceeding the yield strength of the polyethylene material.
